# Direct Inhibition of Cellular Fatty Acid Synthase Impairs Replication of Respiratory Syncytial Virus and Other Respiratory Viruses

**DOI:** 10.1371/journal.pone.0144648

**Published:** 2015-12-11

**Authors:** Yamini M. Ohol, Zhaoti Wang, George Kemble, Gregory Duke

**Affiliations:** 3-V Biosciences, Menlo Park, California, United States of America; Washington State University, UNITED STATES

## Abstract

Fatty acid synthase (FASN) catalyzes the *de novo* synthesis of palmitate, a fatty acid utilized for synthesis of more complex fatty acids, plasma membrane structure, and post-translational palmitoylation of host and viral proteins. We have developed a potent inhibitor of FASN (TVB-3166) that reduces the production of respiratory syncytial virus (RSV) progeny *in vitro* from infected human lung epithelial cells (A549) and *in vivo* from mice challenged intranasally with RSV. Addition of TVB-3166 to the culture medium of RSV-infected A549 cells reduces viral spread without inducing cytopathic effects. The antiviral effect of the FASN inhibitor is a direct consequence of reducing *de novo* palmitate synthesis; similar doses are required for both antiviral activity and inhibition of palmitate production, and the addition of exogenous palmitate to TVB-3166-treated cells restores RSV production. TVB-3166 has minimal effect on RSV entry but significantly reduces viral RNA replication, protein levels, viral particle formation and infectivity of released viral particles. TVB-3166 substantially impacts viral replication, reducing production of infectious progeny 250-fold. *In vivo*, oral administration of TVB-3166 to RSV-A (Long)-infected BALB/c mice on normal chow, starting either on the day of infection or one day post-infection, reduces RSV lung titers 21-fold and 9-fold respectively. Further, TVB-3166 also inhibits the production of RSV B, human parainfluenza 3 (PIV3), and human rhinovirus 16 (HRV16) progeny from A549, HEp2 and HeLa cells respectively. Thus, inhibition of FASN and palmitate synthesis by TVB-3166 significantly reduces RSV progeny both *in vitro* and *in vivo* and has broad-spectrum activity against other respiratory viruses. FASN inhibition may alter the composition of regions of the host cell membrane where RSV assembly or replication occurs, or change the membrane composition of RSV progeny particles, decreasing their infectivity.

## Introduction

Respiratory syncytial virus (RSV) is a ubiquitous human pathogen and a leading cause of lower respiratory tract illness (LRTI) in infants, the elderly, the immunocompromised, and individuals with cardiopulmonary disease worldwide (reviewed in [1]). Patients with chronic obstructive pulmonary disease (COPD) are also susceptible to persistent RSV disease, which may exacerbate lung dysfunction [2, 3]. Annually, RSV is estimated to cause 3.4 million episodes of LRTI requiring hospitalization and 60,000 to 199,000 deaths of children under 5 years old, mostly in developing countries [4]. In the United States, the CDC estimates that each year RSV infection causes 132,000 to 172,000 hospitalizations of children < 5 years old, and 177,000 hospitalizations and 14,000 deaths among adults > 65 years old [5].

The development of novel drugs to treat RSV is an important unmet medical need. The sole drug approved for post-infection treatment of RSV is the nucleoside inhibitor ribavirin, but due to its inconsistent efficacy and toxicity to patients and healthcare providers it is not routinely used [1]. The monoclonal antibody Synagis**®** (pavilizumab) is an immunoprophylaxis and only approved for prevention of RSV in high-risk infants, and it must be delivered monthly by intramuscular injection [6]. Although a few direct-acting antivirals (DAA) are in development, rapid emergence of resistant viral mutants has been documented for all [7–9]. One approach to developing drugs that can treat sensitive as well as DAA-resistant viruses and that have an inherent high barrier to the emergence of drug resistant virus is to target host proteins that the virus depends on for replication. In general, host genes have lower mutation frequencies and replication frequencies compared to viruses and, therefore, should be less mutable and reduce the acquisition of drug resistance. Laboratory studies with model host protein targeted inhibitors have demonstrated this high barrier of resistance in RSV [10] and dengue [11]. In addition, inhibition of a host protein used by multiple viruses offers the potential for broad-spectrum activity.

Host cell lipids are essential for completion of the RSV replication cycle. RSV assembly into viral filaments and budding occur at the plasma membrane, and several lines of evidence point to the importance of specialized membrane microdomains called lipid rafts, which are enriched in cholesterol and sphingolipids, in this process. RSV matures at regions of the host plasma membrane that are enriched in the lipid raft protein markers caveolin-1 and GM1, both of which are incorporated into new virions [12–14]. The RSV proteins F, N, P, L, M2-1 and M have been found in lipid raft fractions [15, 16]. Also, F, which mediates viral entry by causing fusion of viral and cellular membranes, can associate with rafts independently of other viral proteins [17–20]. RSV infection alters the lipid composition of rafts; viral filament formation coincides with increased expression of HMG CoA reductase, the enzyme responsible for *de novo* cholesterol synthesis; and cholesterol depletion inhibits viral filament formation [21]. Significantly fewer infectious virions are released by lipid raft-deficient fibroblasts or lung epithelial cells in which rafts have been disrupted by methyl-β-cyclodextrin [22]. Cholesterol depletion also inhibits viral entry, suggesting that RSV virions dock to cholesterol-rich membrane microdomains [23]. In addition, RSV F protein is post-translationally modified by addition of the fatty acid palmitate at a cysteine residue (C550) in its cytoplasmic domain [24]. The role of the cytoplasmic domain of the F protein remains unclear. One study reported that mutation of C550 or deletion of the cytoplasmic domain did not affect F cell-surface expression or fusion [25]. A separate study reported that maintaining C550 but deleting the rest of the cytoplasmic tail caused F to distribute evenly in the plasma membrane instead of into filaments, abolished association with lipid rafts, and significantly reduced infectious progeny production [26].

Because of RSV’s dependence on lipid rafts and palmitoylated proteins, fatty acid synthase (FASN) may play an essential role in the RSV replication cycle. FASN is a central enzyme in the fatty acid biosynthesis pathway that converts the malonyl-CoA, produced from acetyl-CoA by acetyl-CoA carboxylase I (ACC1), into palmitate. Palmitate is utilized both for post-translational modification of specific proteins by protein acyltransferases (PATs), and for synthesis of more complex fatty acids and lipids by multiple downstream enzymes, including acyl-coA synthetases (ACSL), stearyl-coA desaturase (SCD1) and elongases. Silencing or chemical inhibition of FASN has been shown to suppress replication of multiple viruses, including hepatitis C virus (HCV), hepatitis B virus (HBV), and dengue virus [27–29]. FASN interacts with and increases the activity of the hepatitis C virus (HCV) polymerase NS5B [27]. FASN is overexpressed in HBV-transgenic mice, and the dengue viral protein NS3 redistributes FASN to sites of viral replication and increases fatty acid synthesis [28, 29]. Importantly, FASN can be safely modulated. Although FASN is important for embryonic development, adult FASN+/- mice are healthy despite having FASN enzyme activity 35% lower than in wild-type mice [30]. FASN has been silenced by RNAi or chemically inhibited without significant loss of cellular viability [27–29]. Compounds such as (-)-epigallocatechin gallate (EGCG) and a derivative reduce FASN activity in mice without causing weight loss [31, 32]; however, these compounds affect multiple signaling pathways in addition to FASN [33].

We have developed a series of novel and specific FASN inhibitors that can be orally administered, potently reduce palmitate synthesis, and are well tolerated in mice and Sprague-Dawley rats [34, 35]. In this work, we show that FASN inhibition reduces production of RSV A, RSV B, human parainfluenza virus 3 (PIV3), and human rhinovirus 16 (HRV16) infectious progeny. Suppression of FASN inhibits RSV spread, infectivity of virions, and levels of viral proteins and RNA. In addition, oral delivery of our FASN inhibitor in an RSV mouse model demonstrates potent therapeutic and prophylactic anti-RSV activity.

## Results

### FASN inhibition reduces the production of RSV A, RSV B, PIV3, and HRV16 infectious progeny

**Fig 1:**
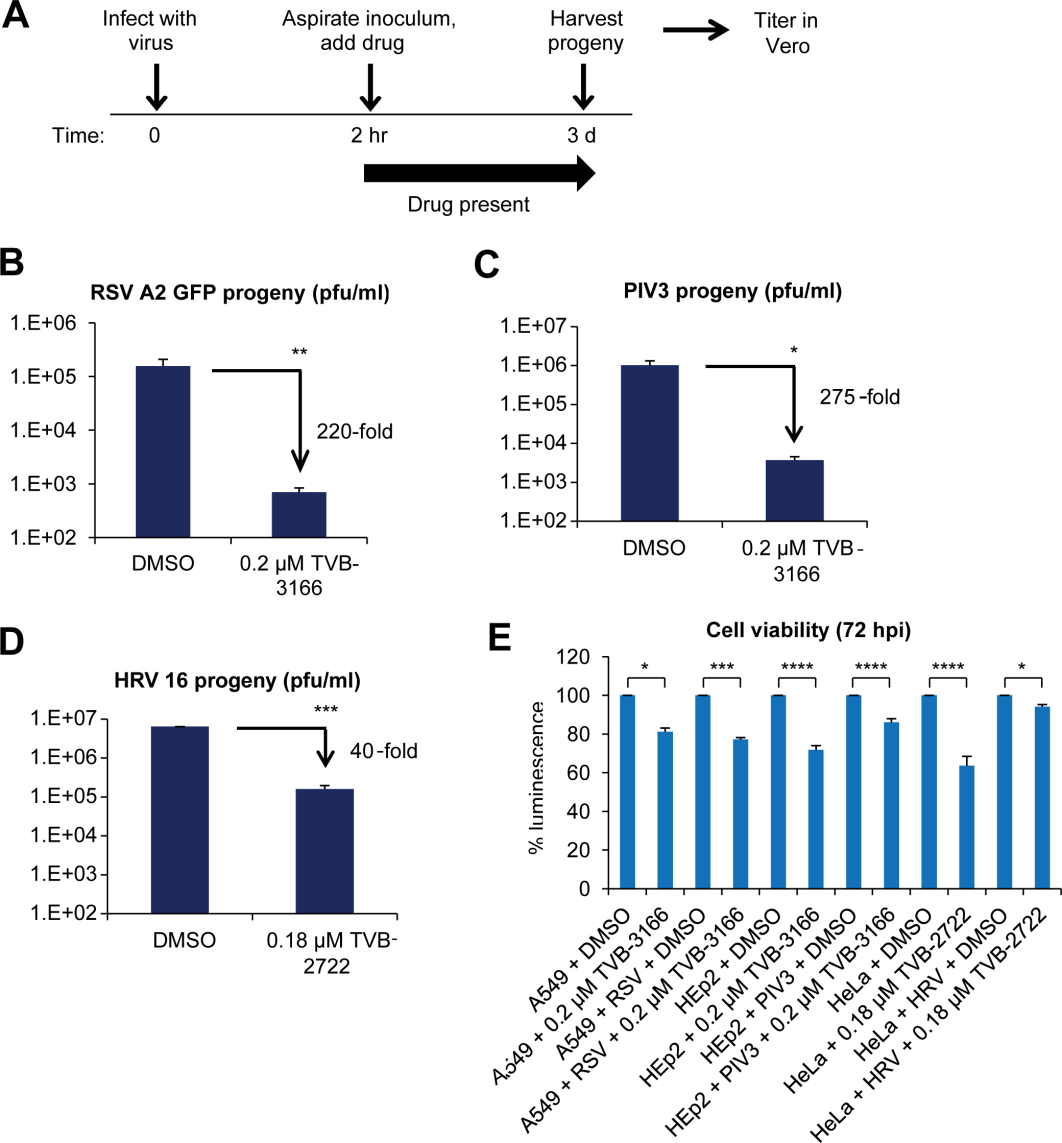
FASN inhibition reduces yield of RSV A, PIV3 and HRV infectious progeny. Experiments were performed as shown in the diagram in (**A**). (**B**) A549 cells were infected with RSV A2 GFP (MOI 0.1). (**C**) HEp2 cells were infected with PIV3 (MOI 0.001). (**D**) HeLa cells were infected with HRV16 (MOI 0.0002). Progeny virus harvested from cells infected for 72 hours in the presence or absence of the FASN inhibitors TVB-3166 or TVB-2722 was used to infect Vero cells. Progeny was titered by automated microscopy (**B**) or plaque assay (**C, D**). (**E**) Infected or mock-infected cells were treated as indicated and cell viability was assessed 72 hours post treatment by Cell Titer Glo assay. Graphs represent the mean ± standard deviation of results from duplicate samples (* = *p*<0.05, ** = *p*<0.01, *** = *p*<0.001).

To test our hypothesis that inhibition of the cellular enzyme, FASN, would suppress RSV replication, we determined the impact of our specific FASN inhibitor, TVB-3166, on RSV production *in vitro*. Virus was added to the cells for 2 hours at 37°C in order to ensure adequate time for viral entry. The viral inocula were then removed and the cells were treated immediately with TVB-3166. The level of infectious viral progeny in the cell media was determined 72 hours post infection (hpi) by automated imaging or plaque assay in Vero cells (Fig 1A). TVB-3166 treatment significantly reduced production of RSV A2 infectious progeny by 220-fold (Fig 1B). After observing the effectiveness of TVB-3166 on RSV, we evaluated the antiviral activity against other respiratory viruses including PIV3 and HRV16. TVB-3166 also reduced production of PIV3 infectious progeny by 275-fold (Fig 1C), and TVB-2722, a structurally closely related FASN inhibitor in the same chemical series, reduced HRV16 infectious progeny by 40-fold (Fig 1D). Preliminary time course experiments measuring RSV A2, PIV3, and HRV16 infection daily starting at 24 hpi indicated that the effect of the FASN inhibitors was most pronounced at 72 hpi, based on which we chose 72 hpi as the most informative time point (data not shown). We also tested multiple doses of TVB-3166 and TVB-2722 on RSV A2, PIV3 and HRV16 and found that fold changes varied in a dose-dependent manner (data not shown). Further, the inhibitors were active in all three cell lines used–A549, HEp2, and HeLa–and the viability of infected cells treated with inhibitor was 80% or higher relative to those treated with vehicle (Fig 1E). We hypothesize that percent cell viability for drug-treated PIV3- or HRV-infected cells appears to be higher than for drug-treated mock-infected cells because drug treatment may protect the infected cells from the cytopathic effect of these viruses by reducing the viral load, or viral infection may upregulate cellular lipid metabolic pathways, blunting the effects of FASN inhibition on cell viability.

**Fig 2:**
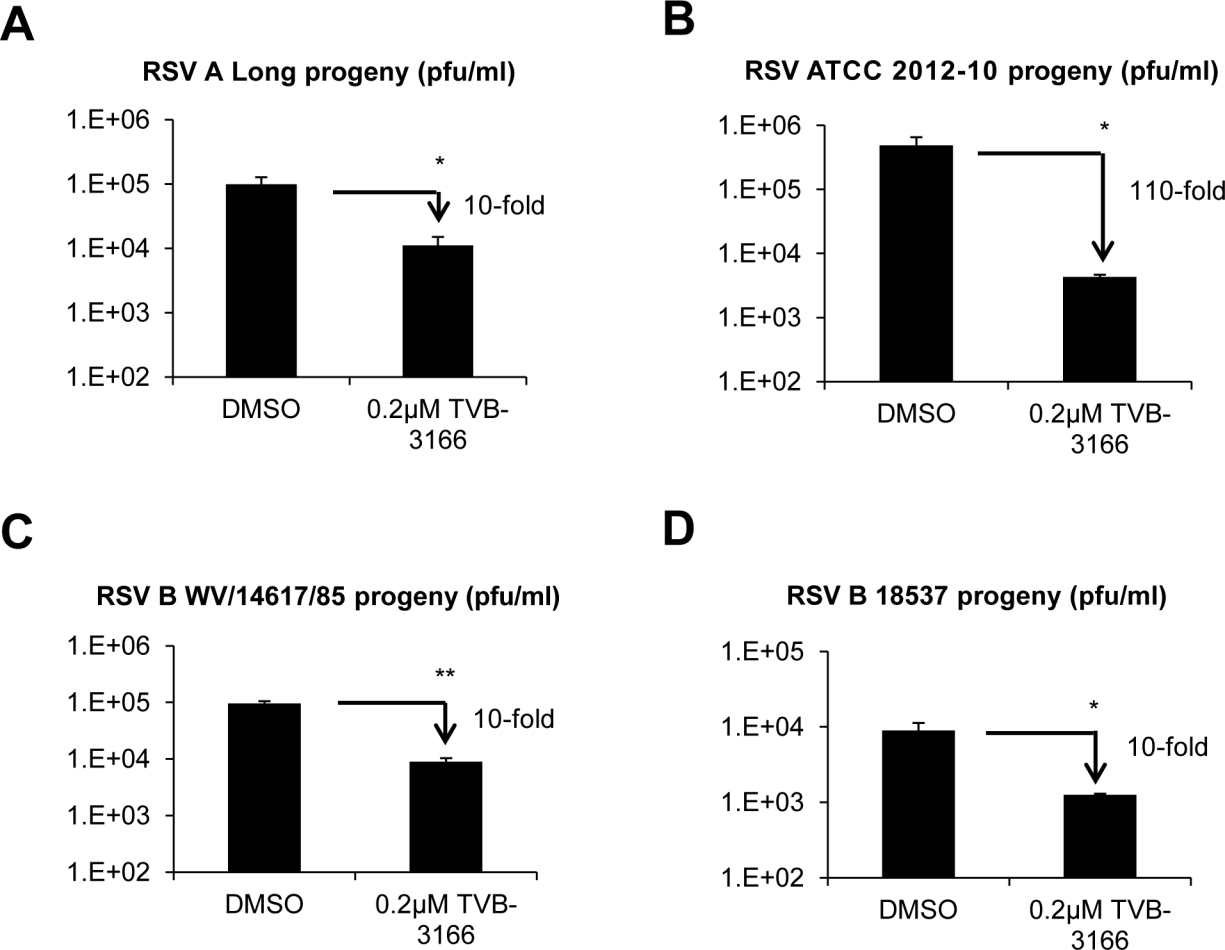
FASN inhibitors reduce infectious progeny of multiple RSV strains. A549 cells were infected at MOI 0.1 with RSV strains A Long (**A**), ATCC 2012–10 (**B**), B-WV/14617/85 (**C**), or B 18537 (**D**). Progeny virus harvested from cells infected for 72 hours in the presence or absence of the FASN inhibitor TVB-3166 was used to infect Vero cells. Progeny was titered by plaque assay. Graphs represent the mean ± standard deviation of results from duplicate samples (* = *p*<0.05, ** = *p*<0.01).

Of the three respiratory viruses we tested, we chose to focus on RSV as it accounts for a significant percentage of severe respiratory illness in humans. We studied the effect of TVB-3166 treatment on infectious progeny production of two RSV A strains (Long and 2012–10, a recent clinical isolate), and two RSV B strains (WV/14617/85 and 18537). FASN inhibition reduced infectious progeny of all four strains by 10- to 110-fold (Fig 2A–2D). RSV A2, A Long, B WV/14617/85, and B 18537 have been extensively passaged under dissimilar culture conditions and may have adapted differently to different cells, and thus have variable susceptibility to host protein-targeted inhibitors such as a FASN inhibitor; however, the molecular basis of these differences cannot be addressed in the current study. The RSV ATCC 2012–10 strain may be more predictive for inhibitor efficacy as it is a more recently isolated and a less passaged clinical strain.

### Anti-RSV activity of TVB-3166 is mediated by palmitate reduction

**Fig 3:**
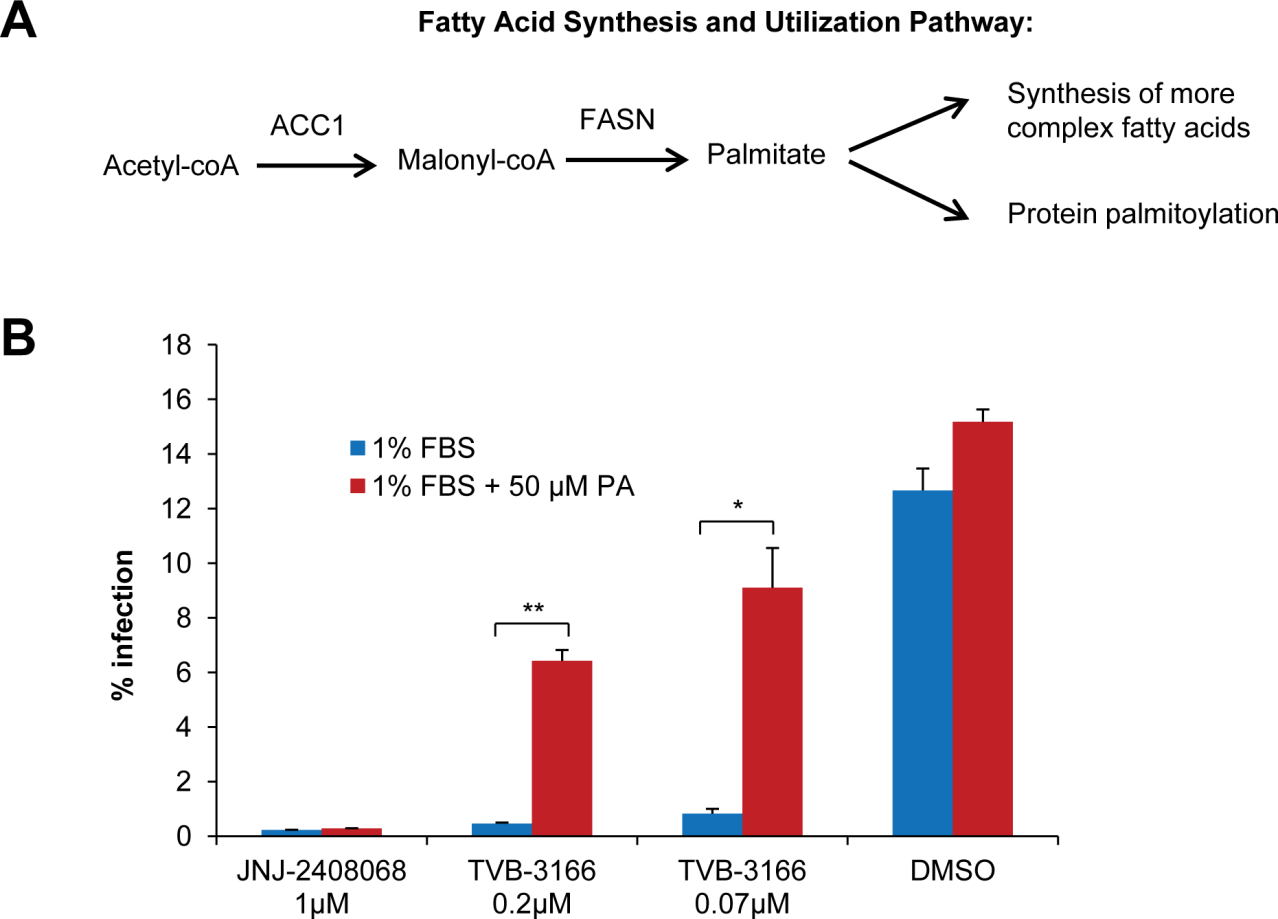
Anti-RSV activity of TVB-3166 is mediated by palmitate reduction. The fatty acid synthesis and utilization pathway is shown in (**A**). (**B**) A549 cells were infected with RSV A2 GFP (MOI 0.03) followed by treatment with TVB-3166 or the RSV fusion inhibitor JNJ-2408068 in the presence or absence of 50 μM palmitate-BSA (PA). Infection levels were quantified 72 hours post infection by automated microscopy. Graphs represent the mean ± standard deviation of results from duplicate samples (* = *p*<0.05, ** = *p*<0.01).

FASN converts the ACC product, malonyl-CoA, into palmitate, which is used for protein palmitoylation by PATs and for synthesis of more complex fatty acids and lipids by enzymes including SCD1 and ACSL (Fig 3A). To verify whether the antiviral activity of TVB-3166 occurs due to reduction of palmitate, we added exogenous palmitate to RSV-infected, TVB-3166-treated cells. Although free palmitate concentrations in human plasma are typically ~100 μM [36], preliminary experiments performed at palmitate concentrations up to 100 μM indicated that 50 μM palmitate provided the best balance between rescue and toxicity in A549 cells. Supplementation with 50 μM palmitate partially restored the replication of RSV in TVB-3166 treated cells; viral infection levels increased over 10-fold compared to un-supplemented cells (Fig 3B). In contrast, addition of exogenous palmitate did not rescue viral growth in cells treated with the fusion inhibitor, JNJ-2408068. Further, TVB-3166 inhibited viral infection at a dose of 0.07 μM, similar to its IC50 in an *in vitro* assay utilizing partially purified FASN (0.05 μM). These data indicate that TVB-3166 inhibits viral growth by an on-target inhibition of FASN activity.

### Inhibition of FASN by TVB-3166 reduces RSV spread and intracellular levels of RSV protein and RNA

**Fig 4:**
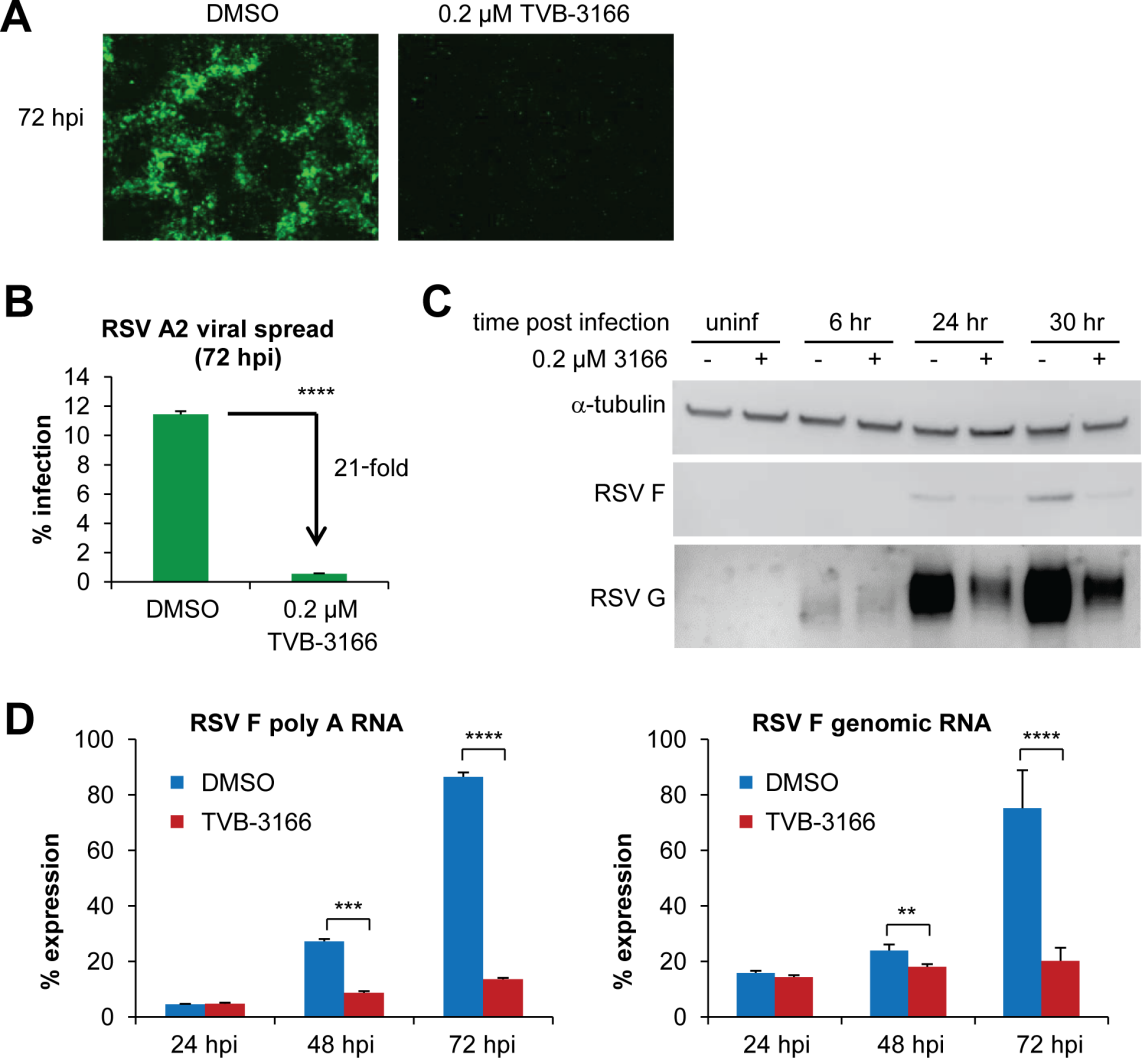
Inhibition of FASN by TVB-3166 reduces RSV spread and intracellular levels of RSV protein and RNA. A549 cells were infected with RSV A2 GFP at MOI 0.1 (**A, B, D**) or MOI 2 (**C**), then treated with DMSO or TVB-3166, and harvested at the indicated times post infection. (**A**) Cells were fixed and imaged at 10X magnification by fluorescent microscopy. (**B**) Percent infection was calculated using automated imaging. (**C**) RSV F and G protein levels were detected by Western blot. (**D**) RSV F RNA levels were determined by qPCR after reverse transcription with oligo-dT or genomic primers and normalized to -actin. qPCR was performed in duplicate from duplicate samples and all C(t) values were normalized to the lowest of the four DMSO-72h C(t) values. Graphs represent the mean ± standard deviation of results from four samples (** = *p*<0.01, *** = *p*<0.001, **** = *p*<0.0001).

We next chose to further characterize TVB-3166 inhibition of RSV by determining which step of the viral life cycle was affected. We observed that viral spread was highly restricted after TVB-3166 treatment, limiting infection to single cells (Fig 4A). Quantitative imaging showed that 11.5% of vehicle-treated cells were infected 72 hours post inoculum addition compared to 0.5% of TVB-3166 treated cells, amounting to a 20-fold reduction in viral infection (Fig 4B). In addition, FASN inhibition reduced the levels of RSV F poly-A (message) RNA and genomic RNA 48–72 hours post infection (Fig 4D), and of RSV F and G proteins at 24 and 30 hours post infection (Fig 4C). We used a high MOI (MOI 2) for the Western blot experiments (Fig 4C) to ensure that most cells were infected and would yield levels of RSV proteins detectable by Western blotting. However, at MOI 2 cells do not survive to 48 hpi due to the cytopathic effect of RSV. Therefore we used a lower MOI (MOI 0.1) for the quantitative PCR experiments (Fig 4D) to allow monitoring of viral genomic RNA and mRNA over a longer time course and measure the effect of TVB-3166 over multiple cycles of replication. The difference between F mRNA and protein levels may be due to a difference in rates of synthesis or degradation of F protein in TVB-3166-treated cells.

### TVB-3166 reduces infectivity of progeny virions

**Fig 5:**
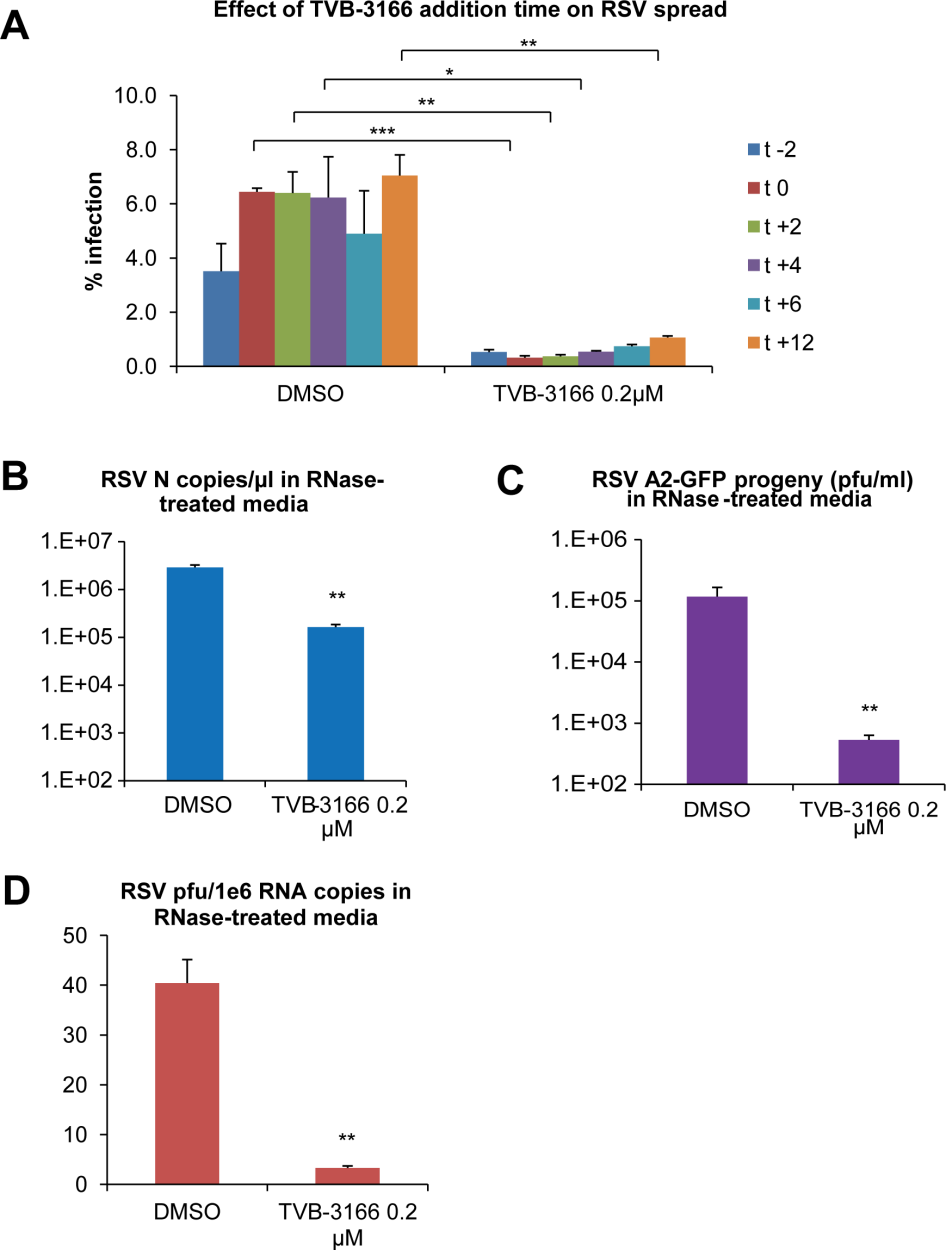
TVB-3166 reduces infectivity of progeny virions. A549 cells were infected with RSV A2 GFP at MOI 0.1 and treated with DMSO or TVB-3166 as indicated. (**A**) Cells were fixed at 72 hpi and percent infection was calculated using automated imaging. (**B-D**) Infected cell media were collected at 72 hpi and treated with RNase A. (**B**) RNase-protected RNA was extracted from cell media and reverse-transcribed with genomic primers, and RSV N RNA levels were determined by qPCR. (**C**) Progeny virus harvested from cell media was used to infect Vero cells and progeny was titered by automated microscopy. (**D**) Infectivity of progeny virions is expressed as PFU per million RNA copies. Graphs represent the mean ± standard deviation of results from duplicate samples (* = *p*<0.05, ** = *p*<0.01, *** = *p*<0.001).

We next investigated the impact of TVB-3166 treatment on various stages of the RSV life cycle. Addition of the inhibitor to cells during the viral attachment period and again immediately post infection (after inoculum was removed) had no additional inhibitory effect compared to treatment immediately post infection, indicating that TVB-3166 did not directly interact with and inactivate the virion (data not shown). Cells pre-treated with TVB-3166 24 hours prior to infection and again immediately post infection had viral infection levels slightly lower than cells treated only immediately post infection (data not shown). However, pre-treatment of cells with TVB-3166 for 24 hours prior to RSV infection followed by removal of the drug after RSV infection did not reduce viral infection levels measured at 48 hpi. In contrast, treatment of cells with TVB-3166 for 24 hours immediately following RSV infection did reduce viral infection levels both at 48 hpi and 72 hpi (data not shown). Together these experiments suggest that TVB-3166 has no significant effect on viral entry. However, TVB-3166 remained effective at reducing viral spread when added up to 12 hours post infection, although a trend toward reduced impact was observed (Fig 5A).

To study the viral particles produced by infected cells in the presence or absence of TVB-3166, we harvested cell media 72 hours post infection and eliminated viral RNA that was not encapsidated by digestion with RNase A. Protected RNA was recovered from one portion of the RNase A-treated media, while the level of infectious progeny virions in another portion was determined by titration on Vero cells. TVB-3166 treatment reduced progeny virus infectivity by ~250-fold (Fig 5C) even though protected RNA levels were only reduced ~10-fold (Fig 5B). Based on protected RNA level, DMSO-treated cells produced 40 PFU of virus per million copies of RSV RNA, compared to 3 PFU of virus per million RSV RNA copies from FASN inhibitor-treated A549 cells, indicating that virions produced in FASN inhibitor treated cells have reduced infectivity (Fig 5D). The difference in the genome/PFU ratio we obtained compared to previously published values [37] is likely due to the different viral strain, cell line, MOI, reverse transcription primers, and quantitative PCR primers, probes, and standards used. Infection at an MOI of 5–10 would be expected to produce the lowest genome/PFU ratio, whereas we infected cells at an MOI of 0.1. Since RSV is known to be a fragile virus that can lose titer even when stored at -80°C, we would expect that in our experiments virus that is not able to infect cells would be inactivated, leading to a higher eventual genome/PFU ratio. Further, although we chose to use the human lung epithelial cell line, A549, as an *in vitro* model for human RSV lung infection, A549 cells are likely less productive than HEp2 cells (a HeLa derivative) in terms of supporting RSV replication.

### Progeny virions from TVB-3166-treated cells fail to replicate in naïve cells

**Fig 6:**
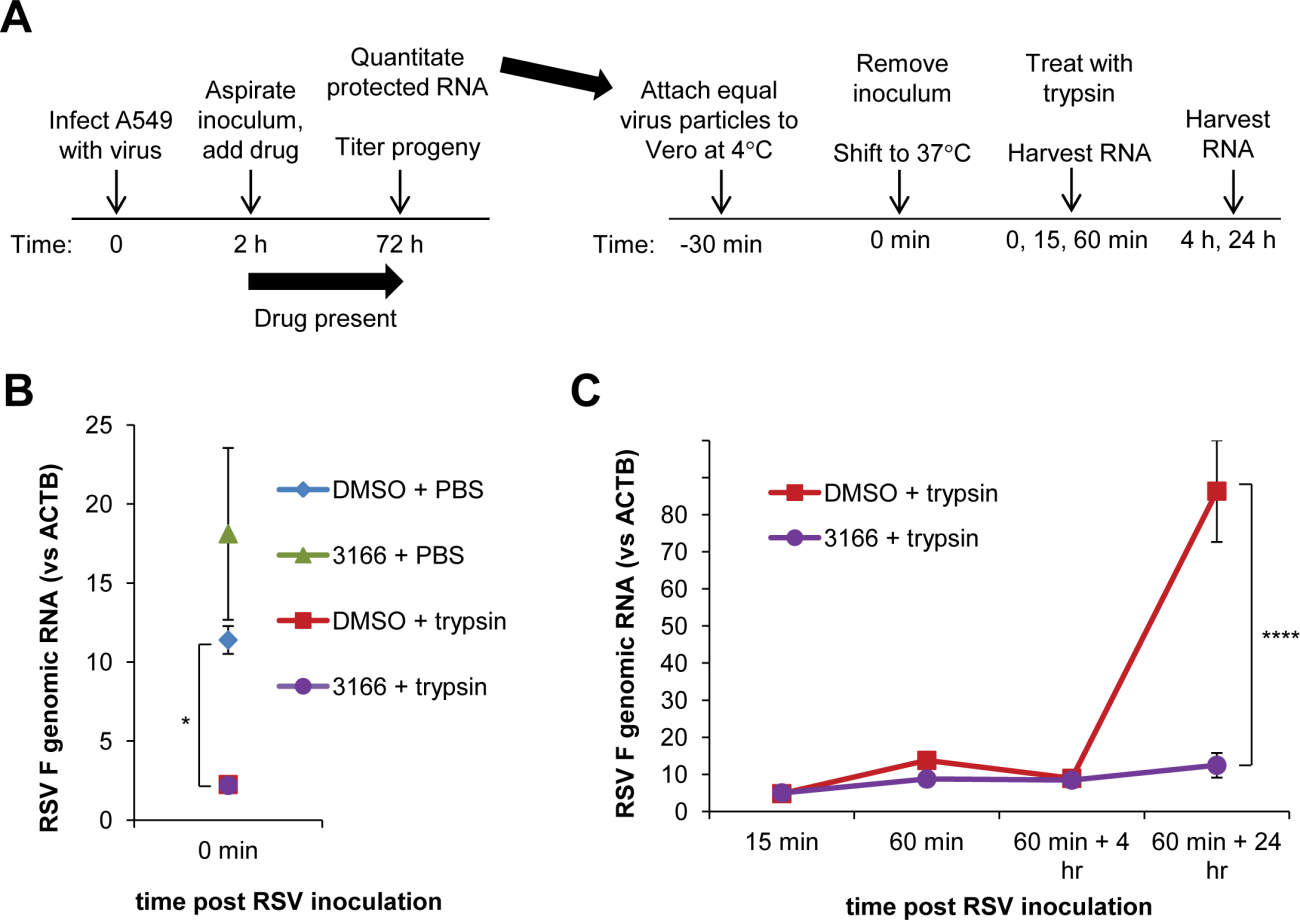
Progeny virions from TVB-3166-treated cells fail to replicate in naïve cells. A549 cells were infected with RSV A2 GFP at MOI 0.3 and treated with DMSO or 0.2 μM TVB-3166 as shown in (**A**). Infected cell media were collected at 72 hpi and treated with RNase A. RNase-protected RNA was extracted from cell media and reverse-transcribed with genomic primers, and RSV progeny particle numbers (RSV N RNA levels) were determined by qPCR. Equal particle numbers of DMSO or TVB-3166 progeny virus were allowed to attach to Vero cells at 4°C, following which cells were shifted to 37°C to allow viral entry, and treated with trypsin to destroy un-internalized virus. Internalized virus was quantitated by qPCR. Viral attachment is shown in (**B**); entry and replication in (**C**). Graphs represent the mean ± standard deviation of results from duplicate samples (* = *p*<0.05, **** = *p*<0.0001).

We evaluated whether the reduced infectivity of RSV virions from TVB-3166-treated cells was due to a failure to attach to, enter, or replicate within cells. We infected Vero cells with equal numbers of viral particles (quantitated by qPCR) from DMSO- and TVB-3166-treated A549 cells (Fig 6A). Vero cells were selected for these experiments since TVB-3166 does not inhibit FASN in these nonhuman primate cells; therefore any differences in entry or replication reflect only changes that occurred during drug treatment in A549 cells, and are not complicated by any potential drug carryover onto Vero cells. Vero cells were inoculated with virus at 4°C to allow virus attachment and viral RNA was quantified by qPCR. Viral RNA levels from cells incubated at 4°C, prior to trypsin treatment, were similar whether the virus was derived from DMSO- or TVB-3166 treated cells, indicating that the viruses attached to a similar extent. Following trypsin treatment to remove un-internalized virus, the viral RNA was significantly reduced from cells exposed to virus from DMSO- or TVB-3166 treated cells (Fig 6B) indicating that very few viral particles had attached in a manner that was no longer sensitive to reversible by protease digestion. Cells were then shifted to 37°C to enable further entry, following which they were treated with trypsin to destroy un-internalized virus and monitored over time. Viral RNA levels remained low and similar for 5 hours after infection, but 25 hours post infection viral RNA levels were 9-fold higher in cells infected with progeny from DMSO-treated cells than from TVB-3166-treated cells (Fig 6C). These data suggest that virions produced from TVB-3166-treated cells had no defect in attachment or entry but were unable to uncoat and/or replicate efficiently.

### TVB-3166 exhibits both prophylactic and therapeutic anti-RSV effects in BALB/c mice

**Fig 7:**
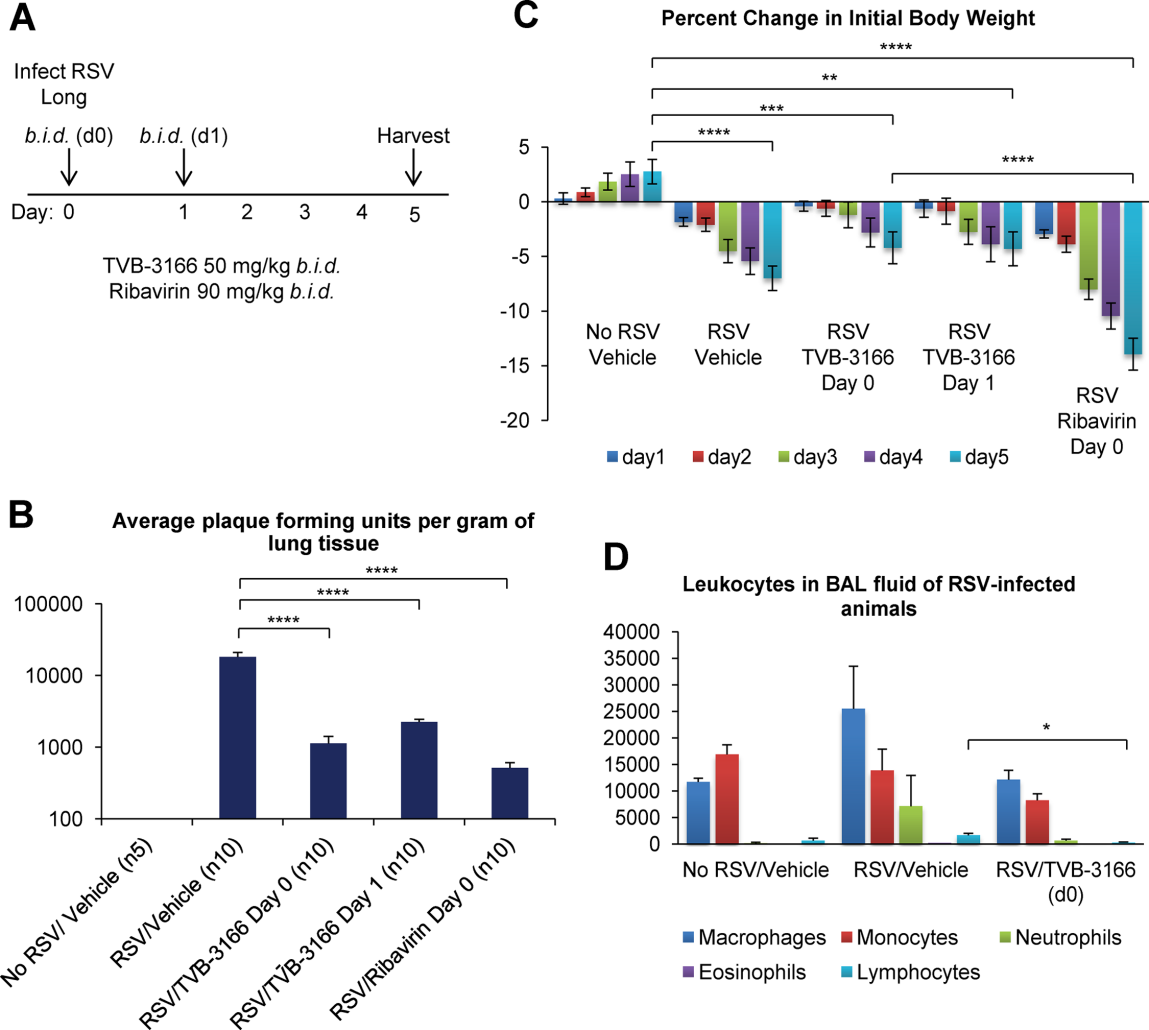
TVB-3166 exhibits both prophylactic and therapeutic anti-RSV effects in BALB/c mice. (**A**) Twice daily treatment with TVB-3166 (p.o.), Ribavirin (i.p.) or drug vehicle (p.o.) was begun 2 hours after intranasal inoculation of mice with 5.5 x 10^5^ PFU of RSV Long. One group received vehicle on day 0 and TVB-3166 on subsequent days. Lungs and broncheoalveolar lavage (BAL) fluid were harvested five days post infection. (**B**) Lung titers were determined by plaque assay on HEp2 cells. (**C**) Percent change in initial body weight was measured daily. (**D**) Leukocyte populations in BAL were quantified by differential count. Graphs represent the mean ± standard deviation of results from groups of 5 or 10 animals (* = *p*<0.05, ** = *p*<0.01, *** = *p*<0.001, **** = *p*<0.0001).

To test whether FASN inhibition has an antiviral effect *in vivo*, we infected BALB/c mice with RSV A (Long) by intranasal inoculation, and orally administered 50 mg/kg TVB-3166 twice a day, starting either on the day of infection or one day post infection (Fig 7A). The nucleoside inhibitor ribavirin (90 mg/kg), delivered intraperitoneally, was used as a positive control. All mice were fed normal chow. Animal body weights were monitored daily and lungs and broncheoalveolar lavage (BAL) fluid were collected five days post infection. TVB-3166 therapy starting immediately post infection and one day post infection reduced RSV titers in lungs 21-fold and 9-fold, respectively, while ribavirin therapy starting on the day of infection led to a 46-fold drop in lung titers (Fig 7B). TVB-3166 administered one day before RSV inoculation was also effective (data not shown). RSV N RNA levels in lungs were also lowered by TVB-3166 and ribavirin therapy (data not shown). Interestingly, TVB-3166 treatment caused smaller reductions in animal body weights over the course of the experiment than did ribavirin, suggesting that it is less toxic than ribavirin (Fig 7C). Preliminary experiments showed that treatment of uninfected mice with similar doses of TVB-3166 treatment reduced the level of weight gain but did not cause weight loss. This could be an effect of the drug on metabolism or due to changes in feeding behavior, and could explain why weight gain in drug-treated infected mice was not completely restored to normal (non drug-treated, uninfected) levels despite reduced viral titers. TVB-3166 treatment also reduced lymphocyte infiltration in RSV-infected mouse lungs, but had no significant effect on infiltration of other leukocytes (Fig 7D).

## Discussion

Host factors are promising targets for novel antiviral drugs, as they potentially present a higher barrier to the development of resistance. FASN supports the replication cycle of the flaviviruses HCV and DENV and the orthohepadnavirus HBV, but its role in respiratory virus infection has not been explored [27–29]. We have discovered that enzymatic inhibition of FASN by TVB-3166 has broad-spectrum anti-respiratory virus activity, reducing RSV A, RSV B, PIV3, and HRV16 infectious progeny production without notable cytopathic effects on infected cells. Suppression of FASN inhibits RSV spread, levels of viral proteins and RNA, and infectivity of new virions. Importantly, oral delivery of our FASN inhibitor demonstrates potent therapeutic and prophylactic activity in a mouse model of RSV.

Palmitate, the fatty acid synthesized by FASN, occupies a central role in many cellular pathways. It is a substrate for synthesis of more complex fatty acids that ultimately contribute to production of cholesterol and sphingolipids, which are enriched in lipid rafts [38]. Compounds that inhibit FASN, such as (-)-epigallocatechin gallate (EGCG) and luteolin, markedly reduce lipid rafts and lipid raft-dependent signaling respectively [39, 40]. However, both compounds affect multiple cellular pathways besides FASN and were used at very high concentrations (20 μg/ml EGCG, 25 μM luteolin) [33, 39, 40]. In addition, post-translational modification of host proteins by palmitate targets them to lipid rafts, maintaining signaling pathways and raft activity (reviewed in [41]). Palmitoylation of viral proteins promotes binding to membranes, virion fusion and entry into host cells, and release of viral particles (reviewed in [42]).

FASN inhibition had no significant effect on RSV entry but reduced levels of viral proteins and RNA in infected cells by approximately 50%, suggesting that viral replication, protein translation, or protein stability were affected. Since RSV polymerase activity and polymerase complex proteins N, P, L, M2-1 and M associate with cytoplasmic inclusion bodies and lipid rafts, viral replication could be affected by perturbation of rafts or membranes associated with inclusion bodies [16, 43]. Another possibility is that FASN blockade leads to degradation of RSV proteins. Inhibition of FASN alters the expression of several ubiquitin ligases and ubiquitin conjugation enzymes in breast cancer cells, and stimulates ubiquitination and degradation of PI3K substrates in ovarian cancer cells [44, 45].

However, the most striking impact of FASN inhibition was on infectious progeny production and its infectivity. One possibility is that viral filament assembly and/or budding is disrupted in TVB-3166-treated cells. But this is unlikely, since protected RNA copy number (a surrogate measure for viral particle number) was only reduced 10-fold, indicating that viral particles were being released. A second hypothesis is that lowered palmitate synthesis due to FASN inhibition results in decreased palmitoylation of RSV F protein, negatively affecting its activity. Mutation of the F palmitoylation site, C550, does not affect fusion [25]. However, this experiment was performed using plasmids transfected into 293T cells, so the effect on attachment or entry of live virus is not known. A third, and likely, possibility is that the virions produced are defective in attachment, entry or replication in naïve cells. A similar phenotype was observed when cholesterol was extracted by methyl-β-cyclodextrin (MBCD) from RSV-infected A549 cells, thus disrupting lipid rafts [22]. RSV virions released from untreated versus MBCD-treated cells were indistinguishable in number and protein composition, but those released from MBCD-treated cells had markedly impaired infectivity and lower associated cholesterol. We observed that virions from FASN inhibitor-treated cells are able to attach to and fuse with naïve cells with wild-type kinetics, but are defective at a later stage, possibly uncoating or replication. Perhaps FASN blockade alters the composition of the RSV envelope by reducing the lipids available for incorporation or by perturbing areas of the host cell membrane from which budding occurs, and the resulting virions are unable to uncoat efficiently. Alternatively, FASN inhibition during the initial infection could perturb viral replication, affecting protein composition of the virions, which then replicate less productively in naïve cells. Lipidomics analysis of mock- and RSV-infected A549 cells suggested that RSV infection slightly increased levels of some *de novo* synthesized fatty acids while TVB-3166 decreased lipogenesis in both mock- and RSV-infected cells (data not shown).

The inhibitory effect of TVB-3166 treatment can largely be reversed by addition of artificially high levels of palmitate, the product of the FASN enzyme, indicating that the antiviral activity of this compound is indeed a consequence of reduced palmitate synthesis. The lack of complete rescue by exogenous palmitate suggests that RSV may preferentially utilize a subcellular pool of palmitate, generated by *de novo* synthesis in a particular location, over dietary palmitate. One finding that supports the existence of distinct subcellular pools of palmitate is that the addition of non-toxic doses of exogenous palmitate does not rescue expression of the PPARα target gene ACO following FASN silencing in Hepa1-6 cells [46]. In these cells, after palmitate addition, the levels of FASN phosphorylation and activity differ in membrane and cytoplasmic fractions. In another example, a sub-population of FASN in prostate cancer cells associates with lipid rafts and physically interacts with Cav-1, which must be palmitoylated for the interaction to occur [47]. One way in which RSV could generate a specific subcellular pool of palmitate or efficiently utilize newly synthesized palmitate is to recruit FASN to sites of viral replication, assembly or budding, such as lipid rafts. For example, dengue virus NS3 protein colocalizes with FASN, increasing its activity and redistributing it to sites of virus replication [29]. TVB-3166 is effective in the presence of serum and in mice fed normal chow, in contrast to some reports of FASN inhibition [48, 49].

The discovery of the dependence of RSV on FASN prompts several future experiments. For example, it would be interesting to determine if FASN inhibition alters the lipid composition of RSV virions by performing a lipidomics analysis on purified virions released from TVB-3166-treated versus untreated cells. Secondly, immunofluorescence microscopy could be used to determine whether RSV recruits FASN to viral replication sites, as does dengue virus. In conclusion, we have shown that TVB-3166 has broad-spectrum activity against multiple strains of RSV and against other respiratory viruses (PIV3, HRV16). Its efficacy in an *in vivo* mouse model equals that of ribavirin, the only approved anti-RSV small molecule drug, but it is better tolerated and can be orally administered. Our data indicate that FASN inhibitors thus have considerable potential as novel pan-antiviral drugs to treat severe respiratory illness.

## Materials and Methods

### Ethics statement

Mouse studies were conducted in strict accordance with the recommendations in the Guide for the Care and Use of Laboratory Animals of the National Institutes of Health and their applicable standard operating procedures for animal handling, including euthanizing when necessary to alleviate suffering. The protocol was approved by Aragen’s Institutional Animal Care and Use Committee (IACUC) (Protocol number: SA-003-A).

### Cells, viruses and inhibitors

Vero, HEp2 and HeLa cells were acquired from American Type Culture Collection (ATCC), and A549 cells were provided by Dr. Ari Helenius (ETH Zurich). Cells were cultured in standard growth media with 10% fetal bovine serum (FBS). RSV A (Long strain), RSV B WV/14617/85, PIV3 (strain C 243) and HRV 16 were purchased from ATCC, and RSV A2 GFP was a gift of Dr. Hong Jin (Medimmune, Mountain View, CA). RSV A2 GFP was constructed essentially as described for the RSV A2 lacZ virus (designated as A-lacZ), except that the GFP gene was cloned instead of the lacZ gene [50]. Preliminary experiments comparing the replication kinetics of RSV A2 GFP and RSV A2 in A549 cells showed that at 3 different MOIs (0.1, 0.3, 0.5) the two viruses grew with very similar kinetics (S1 Fig). Infected cells were fixed at 24, 48 and 72 hpi, immunostained with anti-RSV F antibody, and % infection was quantified by automated imaging. TVB-3166 and TVB-2722 were synthesized at Pharmaron (Beijing, China), JNJ-2408068 was synthesized at BioFocus (South San Francisco, CA), and Ribavirin was purchased from Sigma (St. Louis, MO).

### Viral infection, viral spread, viral progeny titration, and cell viability assays

Cells were seeded into 24-well plates 24 hr before virus infection. Virus was added in OptiMEM and incubated at 37°C for 2 hr, unless otherwise indicated. The inoculum was replaced with standard growth media containing 1% FBS and inhibitors in 0.5% DMSO (final concentration). To measure RSV A2 GFP spread, infected A549 cells were fixed 72 hr post infection with 4% formaldehyde containing 2 μl/ml Hoechst 33342 (Sigma). Images were captured using 10X magnification, and nuclear counterstaining by Hoechst 33342 was used to verify that the GFP staining was cell-associated and due to viral infection (S2 Fig). Automated microscopic imaging and image analysis were performed using an ImageXpress Micro high content microscope and MetaXpress software, respectively (Molecular Devices, Sunnyvale, CA). Progeny titers in pfu/ml were calculated by multiplying the percent infection values obtained using automated microscopy by the number of cells per well, inoculum dilution factor, and inoculum volume factor. We performed parallel plaque assays and found no significant difference in the titer obtained using plaque assays versus automated microscopy. However, we have observed that the passage number of the A549 cells used for these experiments affects the replicative ability of RSV. While we have standardized the number of passages that are acceptable, there is still some variability within the usable passage range. For progeny titration of RSV A2 GFP, RSV B, PIV3 and HRV16, media from infected cells was collected at 72 hpi. Media containing RSV A2 GFP virions was used to infect Vero cells, which were imaged and analyzed using automated microscopy as described. Preliminary experiments showed that TVB-3166 does not inhibit FASN in Vero cells, which are highly permissive for RSV replication. RSV (all strains), PIV3 and HRV16 infectious progeny in media were titered by plaque assay on Vero, HEp2 and HeLa cells, respectively. Cell viability was assessed by Cell Titer Glo luminescent assay (Promega, Madison, WI), and each drug-treated cell viability value was calculated relative to its corresponding DMSO-treated cell viability value, which was set at 100%.

### Western blotting

A549 cells infected with RSV A2 GFP (MOI 2) were lysed in RIPA buffer containing protease inhibitors (Pierce), and lysates were quantitated by BCA assay (Pierce, Rockford, IL). Proteins were visualized by immunoblotting with RSV polyclonal antibody (Abcam, Cambridge, MA) and monoclonal antibodies to α-tubulin (Cell Signaling, Danvers, MA) and RSV F (Abcam).

### Quantitative PCR

A549 cells were infected with RSV A2 GFP (MOI 0.1). Intracellular RNA was extracted using an RNeasy kit (Qiagen, Valencia, CA). Media was treated with 140 U/ml of RNase A (Qiagen), which was subsequently inactivated by treatment with RNAsecure (Life Technologies, Carlsbad, CA). RNase-protected RNA was extracted using a QIAamp MinElute Virus Spin kit (Qiagen). RNA was reverse-transcribed using a High Capacity cDNA Reverse Transcription kit (Life) with included primers or an RSV genome-specific primer: CAATGAACTAGGATATCAAGAC [51]. The cDNA was used to perform quantitative real-time PCR on a LightCycler (Roche, Basel, Switzerland) with the following primers and probes: (i) β-actin: AGCGCGGCTACAGCTTCA (forward), CGTAGCACAGCTTCTCCTTAATGTC (reverse), (FAM) ATTTCCCGCTCGGCCGTGGT (TAMRA) (probe); (ii) RSV F: AGGTCCTGCACCTAGAAGGGGA (forward), TGCTGCAGCTTTGCTTGTTCACA (reverse), (FAM) AGCTGACTACAGCCTTGTTTGTGGA (TAMRA) (probe); (iii) RSV N: GCTCTTAGCAAAGTCAAGTTGAATGA (forward), TGCTCCGTTGGATGGTGTATT (reverse), (FAM) ACACTCAACAAAGATCAACTTCTGTCATCCAGC (TAMRA) (probe). Primers and probes were synthesized at Eurofins (Huntsville, AL). For copy number determination, a standard curve was generated using serial dilutions of plasmids encoding the RSV N or F amplicons (synthesized at DNA 2.0, Menlo Park, CA). For Fig 4D, qPCR was performed in duplicate from duplicate samples and all C(t) values were normalized to the lowest of the four DMSO-72h C(t) values. The DMSO-72h sample with the lowest C(t) value thus represented 100% and all other samples, including the three other replicate DMSO-72h samples, had values lower than 100%. Since the graphs in Fig 4D show the average of the four qPCR replicates, all average values, including the average DMSO-72h values, are less than 100%. The error bars represent the standard deviation within the four qPCR replicates. Initial experiments showed that RSV N and F qPCR primer/probe sets had equivalent efficiencies, yielding virtually identical C(t) values, so they were used interchangeably in subsequent experiments (S3 Fig).

### RSV endocytosis assay

Vero cells were pre-chilled for 10 minutes on ice. RSV inoculum was added and allowed to incubate with cells for 30 minutes on ice. Inoculum was then aspirated and cells were washed twice with PBS. OptiMEM was added and cells were incubated at 37°C. At desired time points cells were treated with 0.1% trypsin or PBS for 5 minutes at room temperature, after which an equal volume of Vero growth medium was added for 5 minutes before being aspirated. Cells were either immediately frozen (to measure viral entry) or OptiMEM was added and cells were incubated at 37°C for desired periods before being frozen (to measure viral replication). RNA was extracted as described above.

### Palmitate addition

5 mM palmitate-BSA was prepared by dissolving palmitic acid (Sigma) in 0.01 M NaOH at 70°C and immediately adding 10% fatty acid-free BSA (Sigma). Growth media was prepared containing 1% FBS, inhibitors and 50 μM palmitate-BSA, and added to infected A549 cells after removing viral inoculum.

### Mouse study

Animal studies were performed at Aragen Bioscience (Gilroy, CA) with 6 to 7 week old female BALB/c mice maintained on normal mouse chow. Percent change in initial body weight was measured daily during the in life phase of the experiment. Four groups of mice were intranasally inoculated with 5.5 x 10^5^ PFU of RSV A (Long). Two hours later, twice daily treatment was begun for three groups with drug vehicle or TVB-3166 (administered by oral gavage), or Ribavirin (injected intraperitoneally). The fourth group received vehicle on day 0 and TVB-3166 on subsequent days. Five days post infection lungs were harvested for virus isolation (n = 10 for infected groups; n = 5 for mock infected vehicle control). Groups subjected to leukocyte analysis had an additional 5 animals from which lungs were harvested for broncheoalveolar lavage (BAL) fluid collection five days post infection. Lungs harvested for virus isolation were homogenized and RSV titers were determined by plaque assay on HEp2 cells. Leukocyte populations in the BAL were quantified by differential count.

### Statistical analysis

For Figs 1–6, graphs represent the mean ± standard deviation of the results from duplicate samples. For Fig 7, graphs represent the mean ± standard deviation of the results from groups of 5 or 10 animals as indicated. Statistical analysis was performed using the Student *t* test for unpaired data and *p* values of <0.05 were considered significant. Data points are labeled with asterisks to indicate *p* values as follows: * = *p*<0.05, ** = *p*<0.01, *** = *p*<0.001, **** = *p*<0.0001.

## Supporting Information

S1 Fig RSV A2 GFP and RSV A2 grow with similar kinetics in A549 cells. A549 cells were infected with RSV A2 GFP or RSV A2 at MOI 0.1, 0.3 or 0.5 and harvested at 24, 48 or 72 hours post infection as indicated. Cells were fixed and imaged by fluorescent microscopy. Percent infection was calculated using automated imaging.
(TIF)

S2 Fig Automated microscopic imaging of RSV A2 GFP-infected A549 cells. A549 cells were infected with RSV A2 GFP at MOI 0.1, treated with TVB-3166 or vehicle as indicated, and fixed 72 hours post infection with 4% formaldehyde containing 2 μl/ml Hoechst 33342 (Sigma). Images were captured by automated microscopy at 10X magnification, using 25 imaging sites per well of a 24-well plate. Nuclear counterstaining by Hoechst 33342 was used to verify that the GFP staining was cell-associated and due to viral infection.
(TIF)

S3 Fig Comparison of C(t) values obtained using RSV F and N qPCR primer/probe sets. A549 cells were infected with RSV A2 GFP at MOI 0.1 and duplicate wells were treated with TVB-3166 or vehicle as indicated. RNA was extracted 24, 48 or 72 hours post infection as indicated, reverse transcribed, and used for qPCR with RSV N or F qPCR primer/probe sets in duplicate.
(TIF)
