# Supplementary figures and images for: Direct Inhibition of Cellular Fatty Acid Synthase Impairs Replication of Respiratory Syncytial Virus and Other Respiratory Viruses
Source: PLoS One. 2015 Dec 11;10(12):e0144648. doi: 10.1371/journal.pone.0144648 (PMC4684246; doi:10.1371/journal.pone.0144648)

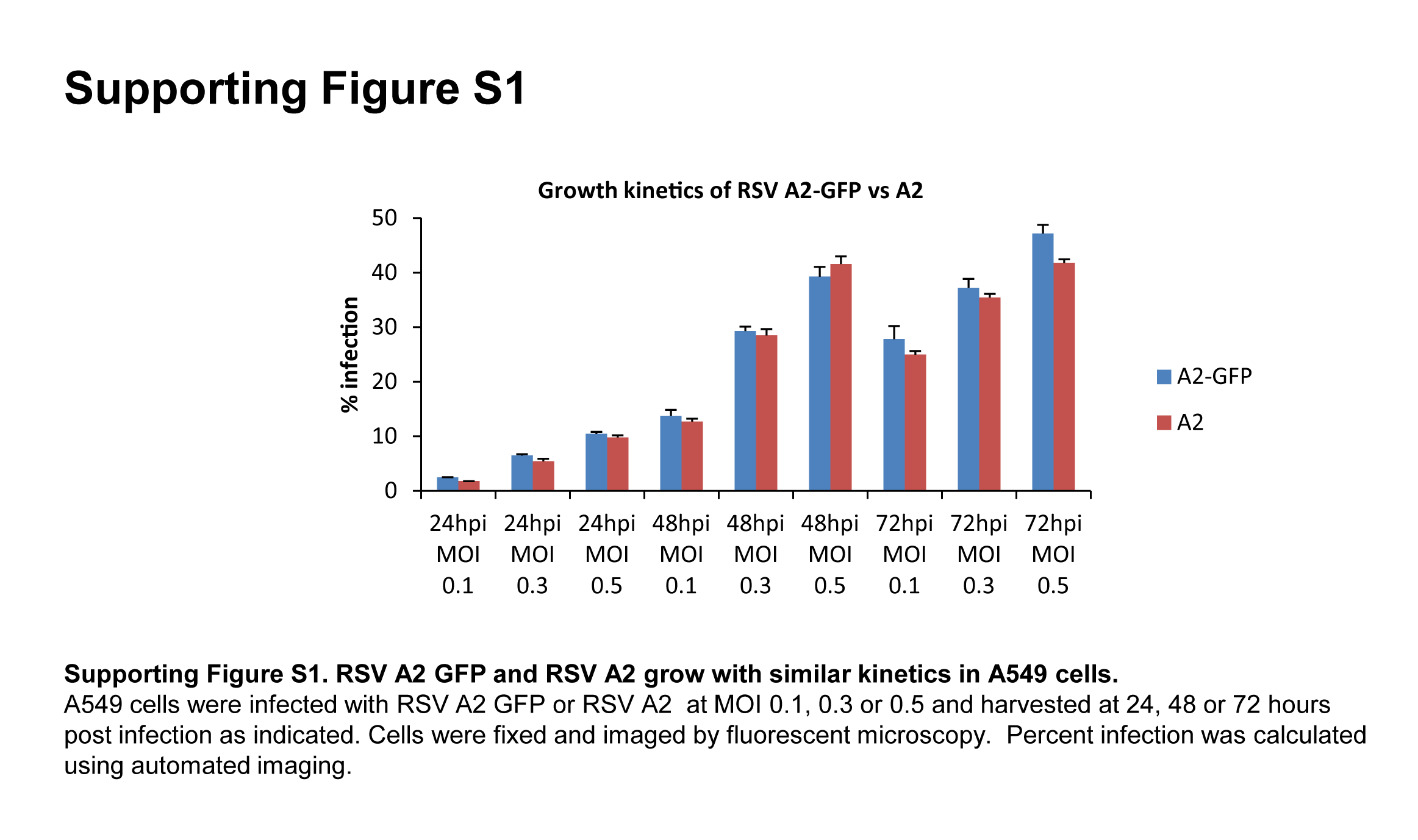

Supplement: S1 Fig — A549 cells were infected with RSV A2 GFP or RSV A2 at MOI 0.1, 0.3 or 0.5 and harvested at 24, 48 or 72 hours post infection as indicated. Cells were fixed and imaged by fluorescent microscopy. Percent infection was calculated using automated imaging. (TIF) [file pone.0144648.s001.tif]

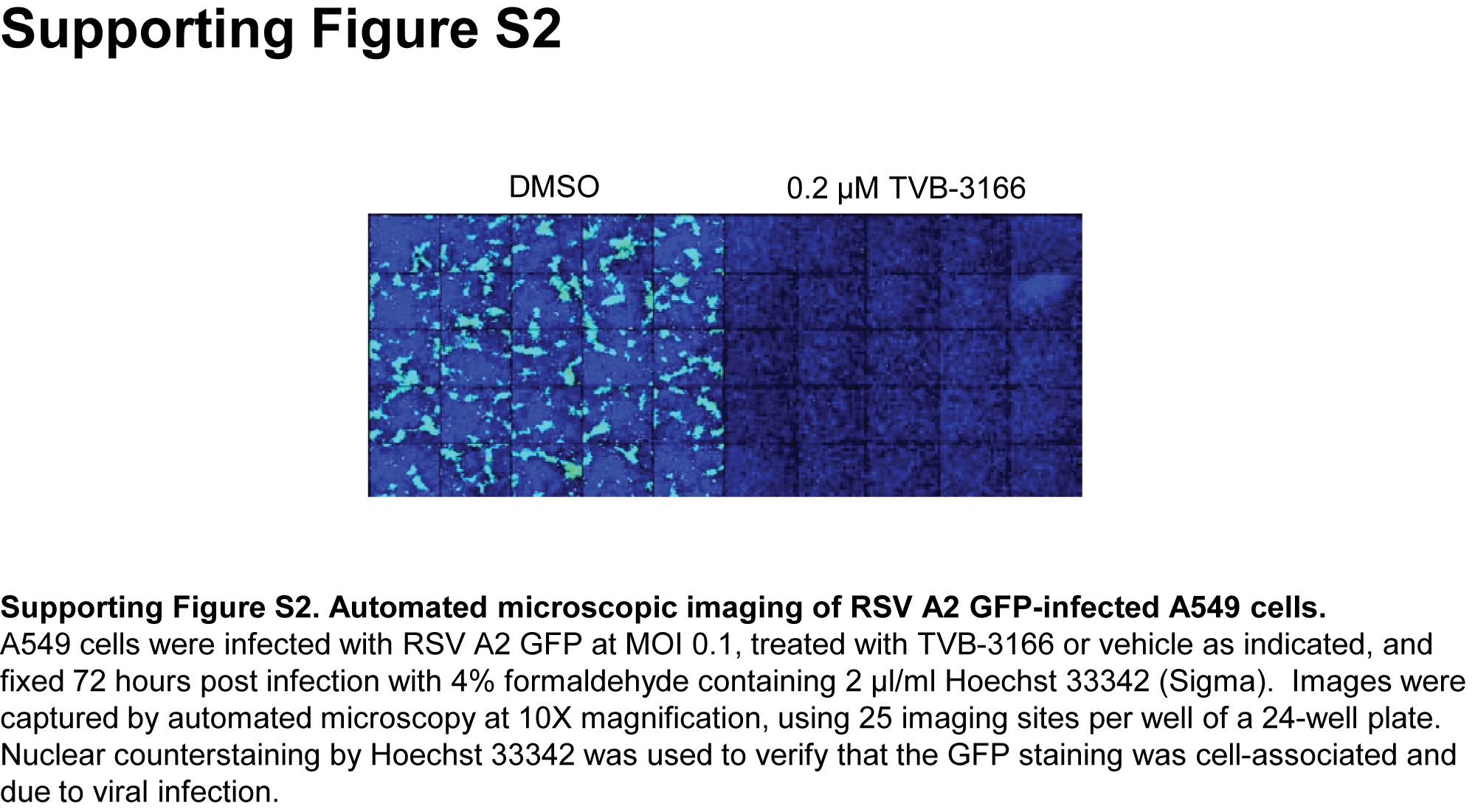

Supplement: S2 Fig — A549 cells were infected with RSV A2 GFP at MOI 0.1, treated with TVB-3166 or vehicle as indicated, and fixed 72 hours post infection with 4% formaldehyde containing 2 μl/ml Hoechst 33342 (Sigma). Images were captured by automated microscopy at 10X magnification, using 25 imaging sites per well of a 24-well plate. Nuclear counterstaining by Hoechst 33342 was used to verify that the GFP staining was cell-associated and due to viral infection. (TIF) [file pone.0144648.s002.tif]

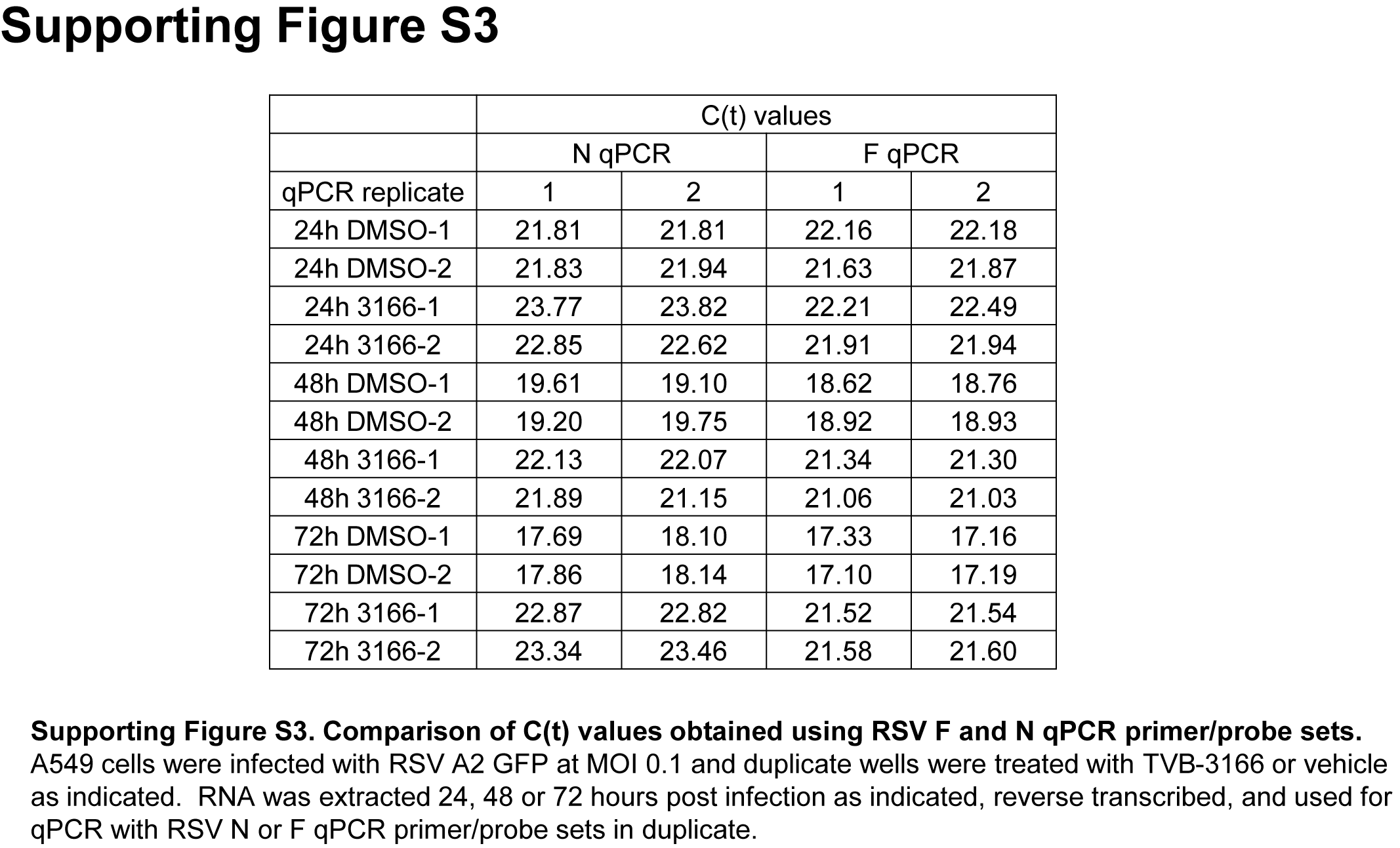

Supplement: S3 Fig — A549 cells were infected with RSV A2 GFP at MOI 0.1 and duplicate wells were treated with TVB-3166 or vehicle as indicated. RNA was extracted 24, 48 or 72 hours post infection as indicated, reverse transcribed, and used for qPCR with RSV N or F qPCR primer/probe sets in duplicate. (TIF) [file pone.0144648.s003.tif]
